# Projected increase in amyotrophic lateral sclerosis from 2015 to 2040

**DOI:** 10.1038/ncomms12408

**Published:** 2016-08-11

**Authors:** Karissa C. Arthur, Andrea Calvo, T. Ryan Price, Joshua T. Geiger, Adriano Chiò, Bryan J. Traynor

**Affiliations:** 1 Neuromuscular Diseases Research Section, Laboratory of Neurogenetics, National Institute on Aging, National Institutes of Health, Bethesda, Maryland 20892, USA; 2 The Commonwealth Medical College, Scranton, Pennsylvania 18509, USA; 3 'Rita Levi Montalcini' Department of Neuroscience, University of Torino, Turin 10124, Italy; 4 Neuroscience Institute of Torino, Turin 10124, Italy; 5 Statistical Genetics Group, Laboratory of Neurogenetics, National Institute on Aging, National Institutes of Health, Bethesda, Maryland 20892, USA; 6 Neurodegenerative Diseases Research Unit, Laboratory of Neurogenetics, National Institute of Neurological Disorders and Stroke, National Institutes of Health, Bethesda, Maryland 20892, USA; 7 Brain Science Institute, Department of Neurology, Johns Hopkins University, Baltimore, Maryland 21205, USA

## Abstract

Although amyotrophic lateral sclerosis (ALS) is relatively rare, the socioeconomic significance of the disease is extensive. It is therefore vital to project the epidemiologic trend of ALS. To date, there have been few published studies attempting to estimate the number and distribution of ALS cases in the upcoming years. Here we show that the number of ALS cases across the globe will increase from 222,801 in 2015 to 376,674 in 2040, representing an increase of 69%. This increase is predominantly due to ageing of the population, particularly among developing nations. This projection is likely an underestimate due to improving healthcare and economic conditions. The results should be used to inform healthcare policy to more efficiently allocate healthcare resources.

## Abstract (web summary)

The socioeconomic burden of amyotrophic lateral sclerosis (ALS) is high, but the projected number of cases in the upcoming years is unclear. Here, the authors estimate the number and distribution of ALS cases to 2040, and show that cases are projected to increase, particularly in developing nations.

## Graphical abstract

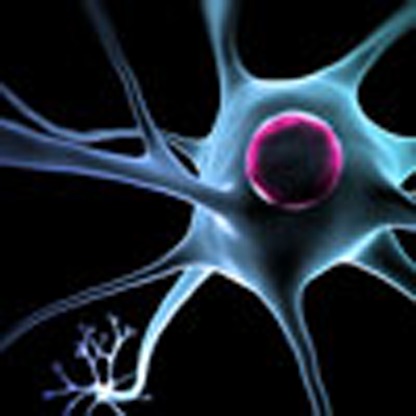

According to the United Nations, the number of individuals in the world above age 60 is expected to increase rapidly. This ageing pattern is especially significant in developing countries, where the proportion of older individuals will increase from about 9% in 2015 to 16% by 2040 (ref. 1). The number of individuals diagnosed with chronic disease has grown due to this trend^2^. For instance, one study found that the number of individuals with Parkinson's disease will double between 2005 and 2030, and the weight of disease will shift from developed nations to developing nations^3^. While similar studies have been conducted on other diseases^4^,^5^, no such study has sought to project the number of amyotrophic lateral sclerosis (ALS) cases.

ALS is the most common adult-onset motor neuron disease. It is characterized by both upper and lower motor neuron degeneration and has a median survival of 2–4 years^6^. The worldwide annual incidence of ALS is about 1.9 per 100,000 (ref. 7), with uniform rates in Caucasian populations and lower rates in African, Asian and Hispanic populations^8^. Although rare, the socioeconomic significance of the disease is substantial^9^,^10^. Accurate projections of ALS case numbers will help guide healthcare policy and the effective allocation of resources.

The objective of our study is to estimate the number of individuals with ALS across the globe in the years 2015 and 2040. To do this, we use previously published data on the incidence of ALS. We show that the number of cases of this fatal neurodegenerative disease will increase by 69% over the next 25 years and that this increase is primarily due to population ageing. We further demonstrate an overall shift in the projected number of ALS cases away from developed countries towards more developing nations.

## Results

### Population and ALS prevalence estimates

We identified ALS incidence studies for 10 countries and geographical regions (China, Europe, Iran, Japan, Libya, New Zealand, Serbia, Taiwan, the United States and Uruguay) that were suitable for analysis. The total population of these countries in 2015 was 2.46 billion, representing 34% of the world population. The total population of the countries above age 20 will increase by 7.7% from 1.89 billion in 2015 to 2.03 billion in 2040.

The prevalence rates of ALS were highest in Uruguay, New Zealand and the United States, and lowest in Serbia, China and Taiwan (Supplementary Tables 1 and 2). The age groups with the highest prevalence rates of ALS were from age 60 to 79.

### Estimates of ALS case numbers in 10 regions

**Figure 1:**
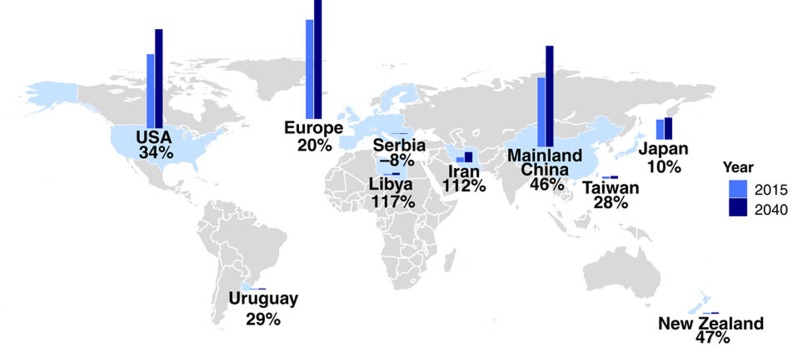
Projected increase in the number of individuals with ALS from 2015 to 2040. The countries studied are shown in light blue. The bars represent the growth in number of cases from 2015 (royal blue) to 2040 (navy). The per cent increase in cases during this time is shown under the country's name. The map was created in R using the packages maps, ggplot2 and ggsubplot (available at https://cran.r-project.org/).

In 2015, there were 45,810 men and 34,352 women diagnosed with ALS in the 10 regions for which incidence rates were available. The projected number of individuals with ALS in 2040 was 60,394 men and 45,299 women. Thus, the total number of individuals with ALS is expected to grow from 80,162 in 2015 to 105,693 in 2040, representing an increase of >31% (Table 1). The geographical distribution of this increase in case numbers is shown in Fig. 1.

**Figure 2:**
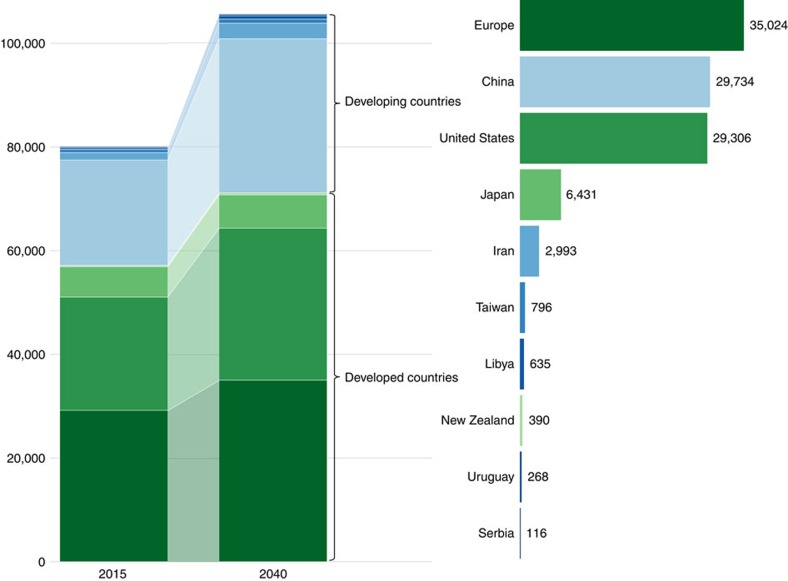
Projected increase in the number of individuals with ALS in developed and developing countries from 2015 to 2040. The bar graph on the left shows the increase in cases in developed (green) and developing (blue) countries from 2015 to 2040. The graph on the right shows the projected number of cases in each country in 2040. The graph was created in R using the packages ggplot2 and ggsubplot (available at https://cran.r-project.org/).

The number of ALS cases will increase substantially in the developing world over the next 25 years (Fig. 2). Notably, developing countries (China, Iran, Libya, Serbia, Taiwan and Uruguay) will see a 50% increase in the number of ALS cases from 2015 to 2040. In contrast, cases in developed countries (Europe, Japan, New Zealand and the United States) will increase by only 24% during the same time period. The net result of this pattern is that the weight of disease will gradually shift from developed countries to developing countries. For instance, in 2015, the developed countries studied accounted for 71% of ALS cases, but this will drop to 67% by 2040. This trend is most notable in Iran and Libya, where the number of individuals with ALS in each country will more than double over this time period.

### Estimate of global ALS case numbers

To estimate the number of cases across the world, incidence rates obtained from countries were applied to the population of each country's respective continent. Incidence rates for the United States were used for North America, Uruguay for South America, Libya for Africa and New Zealand for Oceania. Incidence rates from China, Japan, Taiwan and Iran were weighted according to the size of the study population and applied to Asia. European cases were estimated using incidence rates from the European pooled analysis. By our estimates, the number of cases of ALS in the world will increase from 222,801 in 2015 to 376,674 in 2040, representing an increase of 69%. The largest increase will be seen in Africa with 116%, followed by Asia with 81% and South America with 73%. Meanwhile, the population over the age of 20 in these regions will increase by only 33% over the same time period.

## Discussion

The number of individuals with ALS in the 10 studied countries and regions is expected to increase by nearly one third from 80,162 in 2015 to 105,693 in 2040. A growing population size cannot solely explain this increase. While the number of individuals over the age of 20 living in these countries will increase by only 7.7%, the number of individuals with ALS will increase by 31%. Instead, the observed expansion in ALS cases is most likely due to ageing of the populations of these countries that leads to an increase in the number of individuals in the age groups most at risk of developing ALS (60–79 years of age)^1^.

**Figure 3:**
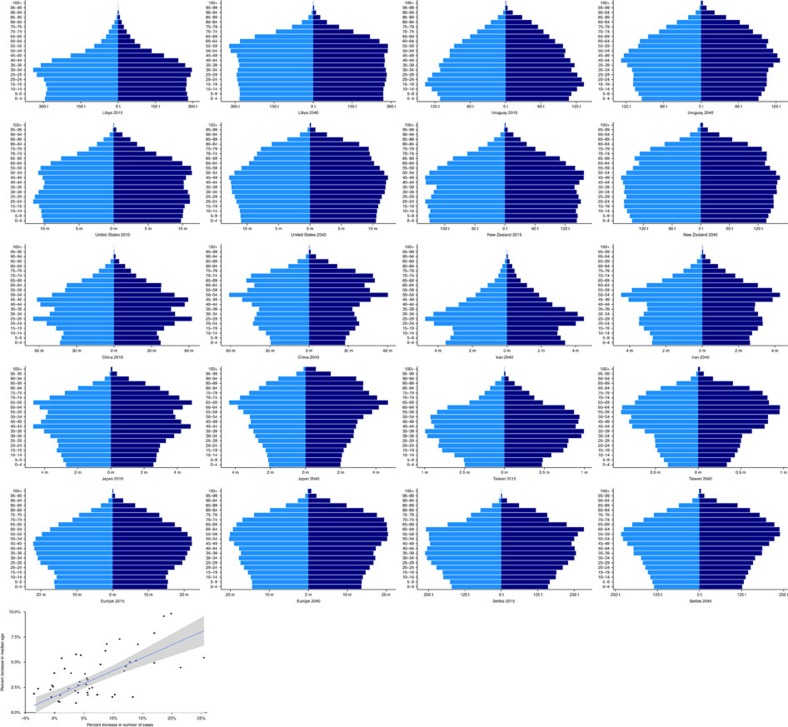
Population growth of the studied countries from 2015 to 2040. The population pyramids show the growth and ageing trends of the populations in the studied countries. Females are located on the right (navy blue) and males on the left (royal blue). The line graph shows a positive correlation between the per cent increase in ALS cases and the per cent increase in median population age from 2015 to 2040 (ref. 37). The graph was created in R using the package ggplot2 (available at https://cran.r-project.org/).

Population ageing may also explain our projection that the weight of ALS will shift from developed countries to developing countries. The adult population in developing countries will increase by only 7.6%, but the number of ALS cases will increase by over 50%. This is most likely due to the rapid ageing of these populations (Fig. 3)^1^. A recent review indicates that the standardized total annual cost per patient with ALS was $69,475 in the United States, $59,018 in Spain, $47,092 in Germany, $21,732 in the Netherlands and $11,251 in Greece^11^. There are currently no studies outlining the cost of ALS in developing countries, and while economic costs are most likely lower in these locations, the increase in ALS cases will place an immense burden on their healthcare systems in the coming decades.

The data for ALS are similar to the projected trend of Parkinson's disease where the number of individuals with this form of neurodegenerative disease will double between 2005 and 2030 (ref. 3). Furthermore, the weight of ALS will continue to shift towards the developing world, a trend also expected with Parkinson's disease. These data mirror the prediction that chronic disease will increase in the coming years due to ageing of the population^2^.

There are several limitations to this study. First, our global estimation that the number of individuals with ALS will increase by more than two thirds is likely conservative. While studies show that incidence rates of ALS tend to remain constant^7^, our projections assume that disease prevalence will also remain constant. However, survival of individuals with ALS will improve as healthcare and economic conditions continue to improve, particularly in developing countries. In addition, while incidence rates over age 80 tend to fall, it is not clear whether this decrease is due to a lower risk of developing ALS in the elderly^12^. It is therefore possible that incidence rates in the elderly are higher than reported, again making the results of this study an underestimate.

Second, our case number calculations rely on published data of country-specific estimates of ALS incidence. While this approach can identify important geographical differences in incidence rates, it also introduces variation due to methodological differences between studies. For example, two studies defined their patient population as those with motor neuron disease rather than ALS (China and Libya)^13^,^14^. Although the majority of motor neuron disease cases are ALS, the incidence rates reported from these studies may be overestimates due to possible inclusion of non-ALS cases. Additionally, one study was published before the El Escorial criteria^14^, and so the cases included in that study may not have been diagnosed accurately. Finally, population-based longitudinal studies are the gold standard for estimating ALS case numbers within a country^15^, but this enhanced type of study was not available for all geographical regions, most notably in Africa, perhaps leading to an underestimate of the global ALS case numbers.

Our projected number of ALS cases should not be taken as an estimate of the global burden of this neurodegenerative disease. The World Health Organization (WHO) defines the burden of disease as years of life lost due to both premature mortality and time lived in states of less than full health^16^. Our study, on the other hand, sought only to calculate the number of individuals throughout the world living with ALS and not to assess their burden of disease. Nevertheless, we maintain that this is a useful addition to the field, as the global burden of ALS has not yet been studied by WHO^17^.

A major strength of this study is that an R code was used to systematically download data from the United States Census International Data Base and calculate the projected number of cases. This code is both reproducible and flexible, and can be used by other researchers to quickly acquire population data and apply it to other diseases. We argue that this type of reproducibility should become the standard in scientific literature^18^,^19^.

In conclusion, the number of individuals with ALS will grow significantly between 2015 and 2040. Furthermore, the weight of disease will shift away from the developed world towards developing countries. Our projection fills a sizeable gap in the scientific literature. Understanding these trends is important to inform healthcare policy and more efficiently allocate local healthcare resources.

## Methods

### Literature search

Age- and sex-specific incidence rates were ascertained from published studies. MEDLINE (via PubMed) was searched for English-language articles published after January 1995, when the El Escorial criteria for diagnosis of ALS were implemented^20^. The MeSH terms ‘amyotrophic lateral sclerosis' or ‘motor neuron disease' along with ‘incidence,' ‘prevalence,' or ‘epidemiology' were used to identify articles. The search was supplemented by examining the reference sections of the retrieved publications. Only articles reporting quantitative incidence rates separated by age group and sex were selected. For countries with multiple available studies, the most recent population-based, observational study was selected.

The population structure of each country from 2015 to 2040 was obtained from the United States Census Bureau International Programs International Data Base^21^. Two recent reviews citing survival rates of ALS were used to identify survival rates for each of the studied regions^22^,^23^.

### Case number calculation

The number of ALS cases was estimated in each country based on the product of (a) the age-specific and sex-specific incidence rates for that particular country, (b) the age-specific and sex-specific population of that country and (c) the median disease duration for that country^24^. This provided comparable results to published prevalence data (see Chiò *et al.*^7^ for review). To make this work reproducible, a computer code was written in R programming to automatically obtain the data from the International Data Base website and then to calculate the ALS case numbers for 2015 and 2040 (ref. 25).

To estimate the number of cases across the world, we applied the incidence and survival rates obtained for each country to their respective continent. If more than one study existed for a continent, the incidence and survival rates were weighted according to the size of the population covered by the study.

### Identified publications

The original search for incidence rates yielded 355 articles. A recent pooled analysis was selected to represent Europe (European Union 28 countries: Austria, Belgium, Bulgaria, Croatia, Cyprus, Czech Republic, Denmark, Estonia, Finland, France, Germany, Greece, Hungary, Ireland, Italy, Latvia, Lithuania, Luxembourg, Malta, the Netherlands, Poland, Portugal, Romania, Slovakia, Slovenia, Spain, Sweden and the United Kingdom)^26^. A total of eight other articles met the full criteria (China, Iran, Japan, New Zealand, Serbia, Taiwan, the United States and Uruguay)^13^,^27^,^28^,^29^,^30^,^31^,^32^,^33^. The search criteria were extended to include an earlier year of publication in order to identify incidence rates for a country in Africa (Supplementary Table 3)^14^. When age ranges of the studies did not match the age ranges of the International Data Base, the given incidence rates were applied across multiple age groups. Seven articles were also identified that gave survival rates for the studied regions^6^,^14^,^27^,^29^,^34^,^35^,^36^.

### Data availability

Data that support the findings of this study were obtained from the United States Census Bureau International Programs International Data Base, which is a public domain resource available at https://www.census.gov/population/international/data/idb/region.php. The R programming code used to analyse these data is freely available at https://github.com/kca5031/ALSCases. All other relevant data are available from the authors on request.

## Additional information

**How to cite this article:** Arthur, K. C. *et al.* Projected increase in amyotrophic lateral sclerosis from 2015 to 2040. *Nat. Commun.* 7:12408 doi: 10.1038/ncomms12408 (2016).

## Supplementary Material

Supplementary Information Supplementary tables 1-3 and supplementary references

## Figures and Tables

**Table 1:** Projected number of ALS cases classified by country and year.

Country	2015		2040	
	Male	Female	Male	Female
*Africa*				
Libya	198	95	433	202
				
*Americas*				
United States	12,656	9,179	17,184	12,122
Uruguay	146	61	193	76
				
*Asia*				
China	12,261	8,068	17,281	12,453
Iran	774	635	1,699	1,293
Japan	3,241	2,625	3,558	2,873
Taiwan	345	277	432	364
				
*Europe*				
EU 28*	15,960	13,248	19,320	15,704
Serbia	74	52	69	48
				
*Oceania*				
New Zealand	154	111	226	165
				
*Total by sex*	*45,810*	*34,352*	*60,394*	*45,299*
				
*Overall total*				
No. of cases/total population	80,162/1.89 billion		105,693/2.04 billion	

^*^EU28, European Union region consisting of 28 countries including Austria, Belgium, Bulgaria, Croatia, Cyprus, Czech Republic, Denmark, Estonia, Finland, France, Germany, Greece, Hungary, Ireland, Italy, Latvia, Lithuania, Luxembourg, Malta, the Netherlands, Poland, Portugal, Romania, Slovakia, Slovenia, Spain, Sweden and the United Kingdom.
