# Supplementary material for: Projected increase in amyotrophic lateral sclerosis from 2015 to 2040
Source: Nat Commun. 2016 Aug 11;7:12408. doi: 10.1038/ncomms12408 (PMC4987527; doi:10.1038/ncomms12408)
Supplement: Supplementary Information — Supplementary tables 1-3 and supplementary references [file ncomms12408-s1.pdf]

Supplementary Information

Supplementary Table 1. Male age-specific prevalence estimates of ALS by country per 100,000 population

Country	Survival Rate	20-24	25-29	30-34	35-39	40-44	45-49	50-54	55-59	60-64	65-69	70-74	75-79	80-84	>85	2015 estimated no. of cases	2040 estimated no. of cases
**Africa**																	
Libya	3.50	0.39	0.39	8.12	8.12	9.94	9.94	28.49	28.49	28.39	28.39	0.00	0.00	0.00	0.00	198	433
**Americas**																	
United States	3.00	0.33	2.37	2.37	4.89	4.89	7.47	7.47	19.26	19.26	30.51	30.51	21.54	21.54	21.54	12,656	17,184
Uruguay	4.33	NA	0.95	0.95	7.49	7.49	12.21	12.21	28.58	28.58	33.25	33.25	28.06	28.06	28.06	146	193
**Asia**																	
China	2.25	0.00	0.00	0.36	2.07	2.21	0.68	1.62	4.07	8.98	4.32	8.10	5.81	3.44	3.44	12,261	17,281
Iran	3.25	0.23	0.00	0.68	1.85	2.63	3.15	6.47	8.68	11.02	7.25	9.13	5.33	7.74	7.74	774	1,699
Japan	2.25	0.00	0.00	0.23	0.23	1.13	1.13	5.40	5.40	12.15	12.15	18.68	18.68	11.48	11.48	3,241	3,558
Taiwan	2.25	0.95	0.88	1.17	1.64	2.90	3.44	4.55	4.95	9.05	10.15	9.32	6.32	2.07	2.07	345	432
**Europe**																	
EU28*	2.39	0.24	0.72	0.72	1.43	3.35	5.26	5.74	13.62	13.38	20.79	25.57	23.18	17.69	4.78	15,960	19,320
Serbia	2.39	0.00	0.00	0.00	0.67	1.72	0.67	3.32	6.38	11.59	5.19	2.13	NA	NA	NA	74	69
**Oceania**																	
New Zealand	2.30	1.15	1.15	1.15	1.15	1.15	1.15	8.51	8.51	24.38	24.38	41.86	41.86	17.48	17.48	154	226
**Total no. of cases / population**																**45,810 / 939 million**	**60,394 / 1.01 billion**

*EU28, region consisting of the 28 European Union countries: European Union 28 countries: Austria, Belgium, Bulgaria, Croatia, Cyprus, Czech Republic, Denmark, Estonia, Finland, France, Germany, Greece, Hungary, Ireland, Italy, Latvia, Lithuania, Luxembourg, Malta, the Netherlands, Poland, Portugal, Romania, Slovakia, Slovenia, Spain, Sweden, and the United Kingdom

Supplementary Table 2. Female age-specific prevalence estimates of ALS by country per 100,000 population

Country	Survival Rate	20-24	25-29	30-34	35-39	40-44	45-49	50-54	55-59	60-64	65-69	70-74	75-79	80-84	>85	2015 estimated no. of cases	2040 estimated no. of cases
**Africa**																	
Libya	3.50	0.00	0.00	2.84	2.84	7.60	7.60	13.16	13.16	7.95	7.95	8.96	8.96	8.96	8.96	95	202
**Americas**																	
United States	2.42	0.00	0.19	0.19	0.87	0.87	7.21	7.21	14.83	14.83	17.93	17.93	13.41	13.41	13.41	9,179	12,122
Uruguay	4.00	NA	0.84	0.84	2.84	2.84	4.20	4.20	11.60	11.60	10.20	10.20	6.52	6.52	6.52	61	76
**Asia**																	
China	2.50	0.00	0.35	0.33	0.55	0.30	1.15	1.95	3.53	3.50	5.88	3.80	2.55	3.25	3.25	8,068	12,453
Iran	5.00	0.00	0.85	1.10	1.25	1.45	4.25	5.30	4.55	5.65	7.40	10.55	4.25	0.00	0.00	635	1,293
Japan	2.50	0.00	0.00	0.25	0.25	1.75	1.75	3.75	3.75	7.75	7.75	12.75	12.75	7.25	7.25	2,625	2,873
Taiwan	2.50	0.53	0.43	0.68	1.83	2.70	3.45	3.25	3.75	6.35	5.08	8.00	4.25	2.60	2.60	277	364
**Europe**																	
EU28*	2.39	0.24	0.96	0.24	0.48	1.91	3.59	5.50	6.69	13.86	16.01	15.54	16.73	9.56	6.21	13,248	15,704
Serbia	2.39	0.67	0.00	0.00	0.00	1.72	1.39	1.24	0.79	7.22	5.19	3.59	NA	NA	NA	52	48
**Oceania**																	
New Zealand	2.30	0.92	0.92	0.92	0.92	0.92	0.92	8.74	8.74	11.50	11.50	27.83	27.83	11.04	11.04	111	165
**Total no. of cases / population**																**34,352/ 949 million**	**45,299/ 1.03 billion**

*EU28, region consisting of the 28 European Union countries: Austria, Belgium, Bulgaria, Croatia, Cyprus, Czech Republic, Denmark, Estonia, Finland, France, Germany, Greece, Hungary, Ireland, Italy, Latvia, Lithuania, Luxembourg, Malta, the Netherlands, Poland, Portugal, Romania, Slovakia, Slovenia, Spain, Sweden, and the United Kingdom

Supplementary Table 3. Epidemiology studies containing age- and sex-specific incidence data used to calculate the projected number of ALS cases

Study	Country/Region	Crude incidence rate per 100,000	Population size
Fong (2005)[1]	China	0.60	6,708,389
Logroscino (2010)[2]	Europe	2.16	23,978,781
Sajjadi (2010)[3]	Iran	0.42	4,559,256
Doi (2014)[4]	Japan	2.20	103,001,116
Radhakrishnan (1986)[5]	Libya	0.89	518,745
Murphy (2008)[6]	New Zealand	3.30	520,280
Alčaz (1996)[7]	Serbia	0.54	1,602,226
Lai (2008)[8]	Taiwan	1.05	22,770,000
McGuire (1996)[9]	United States	1.80	2,559,164
Vazquez (2008)[10]	Uruguay	1.37	3,241,003

Supplementary References

1. Fong, G. C. *et al.* An epidemiological study of motor neuron disease in Hong Kong. *Amyotroph Lateral Scler Other Motor Neuron Disord* **6**, 164-168 (2005).
2. Logroscino, G. *et al.* Incidence of amyotrophic lateral sclerosis in Europe. *J Neurol Neurosurg Psychiatry* **81**, 385-390 (2010).
3. Sajjadi, M. *et al.* Epidemiology of amyotrophic lateral sclerosis in Isfahan, Iran. *Eur J Neurol* **17**, 984-989 (2010).
4. Doi, Y., Atsuta, N., Sobue, G., Morita, M. & Nakano, I. Prevalence and incidence of amyotrophic lateral sclerosis in Japan. *J Epidemiol* **24**, 494-499 (2014).
5. Radhakrishnan, K., Ashok, P. P., Sridharan, R. & Mousa, M. E. Descriptive epidemiology of motor neuron disease in Benghazi, Libya. *Neuroepidemiology* **5**, 47-54 (1986).
6. Murphy, M., Quinn, S., Young, J., Parkin, P. & Taylor, B. Increasing incidence of ALS in Canterbury, New Zealand: A 22-year study. *Neurology* **71**, 1889-1895 (2008).
7. Alčaz, S. *et al.* Epidemiological and clinical characteristics of ALS in Belgrade, Yugoslavia. *Acta Neurol Scand* **94**, 264-268 (1996).
8. Lai, C. H. & Tseng, H. F. Epidemiology and medical expenses of motor neuron diseases in Taiwan. *Neuroepidemiology* **31**, 159-166 (2008).
9. McGuire, V., Longstreth, W. T., Jr., Koepsell, T. D. & van Belle, G. Incidence of amyotrophic lateral sclerosis in three counties in western Washington state. *Neurology* **47**, 571-573 (1996).
10. Vazquez, M. C. *et al.* Incidence and prevalence of amyotrophic lateral sclerosis in Uruguay: a population-based study. *Neuroepidemiology* **30**, 105-111, (2008).
